# Trends in adverse maternal outcomes during childbirth: a population-based study of severe maternal morbidity

**DOI:** 10.1186/1471-2393-9-7

**Published:** 2009-02-25

**Authors:** Christine L Roberts, Jane B Ford, Charles S Algert, Jane C Bell, Judy M Simpson, Jonathan M Morris

**Affiliations:** 1 Clinical and Population Perinatal Health Research, The Kolling Institute of Medical Research, University of Sydney 2006, NSW, Australia; 2 Department of Obstetrics and Gynaecology, Royal North Shore Hospital, St Leonards 2065, NSW, Australia; 3 School of Public Health, University of Sydney 2006, NSW, Australia

## Abstract

**Background:**

Maternal mortality is too rare in high income countries to be used as a marker of the quality of maternity care. Consequently severe maternal morbidity has been suggested as a better indicator. Using the maternal morbidity outcome indicator (MMOI) developed and validated for use in routinely collected population health data, we aimed to determine trends in severe adverse maternal outcomes during the birth admission and in particular to examine the contribution of postpartum haemorrhage (PPH).

**Methods:**

We applied the MMOI to the linked birth-hospital discharge records for all women who gave birth in New South Wales, Australia from 1999 to 2004 and determined rates of severe adverse maternal outcomes. We used frequency distributions and contingency table analyses to examine the association between adverse outcomes and maternal, pregnancy and birth characteristics, among all women and among only those with PPH. Using logistic regression, we modelled the effects of these characteristics on adverse maternal outcomes. The impact of adverse outcomes on duration of hospital admission was also examined.

**Results:**

Of 500,603 women with linked birth and hospital records, 6242 (12.5 per 1,000) suffered an adverse outcome, including 22 who died. The rate of adverse maternal outcomes increased from 11.5 in 1999 to 13.8 per 1000 deliveries in 2004, an annual increase of 3.8% (95%CI 2.3–5.3%). This increase occurred almost entirely among women with a PPH. Changes in pregnancy and birth factors during the study period did not account for increases in adverse outcomes either overall, or among the subgroup of women with PPH. Among women with severe adverse outcomes there was a 12% decrease in hospital days over the study period, whereas women with no severe adverse outcome occupied 23% fewer hospital days in 2004 than in 1999.

**Conclusion:**

Severe adverse maternal outcomes associated with childbirth have increased in Australia and the increase was entirely among women who experienced a PPH. Reducing or stabilising PPH rates would halt the increase in adverse maternal outcomes.

## Background

Maternal deaths in childbirth have declined in high-income countries such that they are now rare occurrences (<10/100,000 livebirths) [1-3]. As mortality has traditionally been used as an indicator of the quality of health care, severe maternal morbidity has been suggested as a better indicator of the quality of maternity care [4-10]. Obstetric haemorrhage is the single most important cause of both maternal mortality and severe morbidity worldwide [4,10-12]. Increasing rates of postpartum haemorrhage (PPH), and maternal deaths attributable to PPH, have been reported in Australia, Canada and the United Kingdom [2,13,14].

Assessment of severe maternal morbidity has relied on intensive methods of data collection in single hospitals or limited populations [15]. Composite measures of severe maternal morbidity based on routinely collected population health data have been used occasionally [4,8]. Composite population-level measures of severe morbidity help overcome problems such as under-ascertainment of individual adverse events and random fluctuations in the component events, and provide an overall count of maternal morbidity in childbirth that is not tied to specific conditions or modes of care [11]. To date however, studies utilising population health data have relied on maternal morbidity measures that have not been validated and have included outcomes that may not be reliably reported [4,8].

The maternal morbidity outcome indicator (MMOI) is a validated measure of severe adverse outcomes associated with childbirth which was developed for use in population health data. [11]. The MMOI is a composite indicator which uses information on diagnoses and procedures from birth and hospital discharge data (see Table 1) which together, accurately and reliably identify women who suffered a severe adverse outcome at the time of childbirth. It is a broad measure of adverse maternal outcome (eg hysterectomy, renal failure) rather than causal conditions (eg haemorrhage, preeclampsia). Although clinical studies may be able to identify 'near miss' morbidity (events where the woman almost died) [6,7,16], population health data lack the clinical detail necessary to draw such a conclusion with confidence. The MMOI identifies adverse outcomes from the severe end of the morbidity spectrum with a broader remit than 'near miss' morbidity [11]. The MMOI was developed as a tool for assessing the quality of maternity care and does not include factors that might be directly linked to service provision, such as intensive care unit admission. The aim of this study is to determine trends in severe adverse maternal outcomes during the birth admission using the MMOI and, in particular, to examine the contribution of postpartum haemorrhage.

**Table 1:** Frequency of diagnoses and procedures contributing to the maternal morbidity outcome indicator (MMOI) during the birth admission 1999–2004

**Indicators of severe maternal morbidity**	Frequency*	**Rate/10 000**
**Morbid events/diagnoses**†	**1340**	
Shock	321	6.41
Uterine rupture	265	5.29
Cardiac failure	225	4.49
Obstetric embolism	194	3.88
Acute renal failure	105	2.10
Major complication of anaesthesia	95	1.90
Psychosis	29	0.58
Status asthmaticus	21	0.42
Status epilepticus	19	0.38
Acute appendicitis	18	0.36
Cerebrovascular accident	16	0.32
**Procedures indicating morbidity**†	**6320**	
Any transfusion‡	4710	94.1
Blood transfusion	4552	90.9
Transfusion of other blood products	612	12.4
*Procedures to control bleeding*		
Evacuation haematoma	306	6.11
Hysterectomy	156	3.12
Dilatation and curettage with GA	155	3.10
Embolisation or ligation of blood vessels	40	0.80
Other interventions to control post-operative bleeding	48	0.96
*Other procedures*		
Assisted ventilation	132	2.64
Repair bladder	113	2.26
Repair ruptured uterus	66	1.32
Cystostomy	64	1.28
Reclose disrupted CS wound	24	0.48
Repair small or large intestine	18	0.36
Dialysis	17	0.34

*women can have more than one diagnosis and/or procedure.

† Total includes diagnoses and procedures with ≤ 10 events including, myocardial infarction, cerebral oedema, peritonitis, disseminated intravascular coagulopathy, sickle cell anaemia with crisis and repair of inverted uterus.

‡ Includes transfusion of blood or blood products (eg platelets, coagulation factors).

GA = general anaesthesia

## Methods

### Study population

The study population included all women who gave birth in New South Wales (NSW) hospitals from 1 January 1999 to 31 December 2004. NSW is the most populous state in Australia with ~6.8 million people and approximately one-third of all Australian births in over 100 hospitals. During the study period only 0.1% of women had home births [17].

### Data sources

The population health data for this study were obtained from two validated, NSW Department of Health computerised datasets: 'birth data' from the Midwives Data Collection and 'hospital data' from the Admitted Patients Data Collection [18]. The birth data are collected in a legislated population-based surveillance system covering all births ≥ 20 weeks' gestation or ≥ 400 g birthweight. Information on maternal characteristics, pregnancy, labour, delivery and infant outcomes are reported by the attending midwife or doctor. Hospital data are a census of all NSW inpatient hospital discharges (public and private); diagnoses and procedures are coded for each admission from the medical records according to the 10th revision of the International Statistical Classification of Diseases and Related Health Problems, Australian Modification (ICD-10-AM) and the affiliated Australian Classification of Health Interventions [19]. The NSW Department of Health performed record linkage of the two datasets, and provided anonymised, linked birth and hospital data for the birth admission. Over 98% of birth records link to a hospital discharge record [18].

Women with any of the diagnoses or procedures that make up the maternal morbidity outcome indicator (MMOI) recorded in their hospital data were considered to have suffered a severe adverse outcome during the birth admission [11]. Data from 21 diagnosis and 20 procedure fields in each medical record were included as this was the maximum number of fields available in 1999. The MMOI does not include factors that predispose women to adverse outcomes, such as preeclampsia and haemorrhage. Instead adverse consequences of these conditions are included, such as acute renal failure, disseminated intravascular coagulopathy and blood transfusions (Table 1).

Information on sociodemographic and pregnancy risk factors for maternal morbidity was obtained from the birth and/or hospital data. Only factors (listed in Tables 2 and 3) that are well and accurately reported in birth and/or hospital data were included in the analysis [20-25]. Birth data provided information on pregnancy and birth characteristics, such as plurality, parity, duration of pregnancy at first antenatal visit, smoking status during pregnancy, labour and delivery characteristics and maternal death. Hospital data provided information on the duration of the hospital stay and on ICD10-AM diagnoses and procedures. Postpartum haemorrhage (PPH) was defined according to ICD10-AM as a haemorrhage of 500 ml or more following vaginal delivery or 750 ml or more following a caesarean delivery resulting in a recorded clinical diagnosis of PPH and identified during the birth hospitalisation from the hospital data [19]. PPH in the hospital data is reported with 74% sensitivity and 99% specificity when compared with the medical record [21]. Only severe PPH (requiring transfusion, procedures to control bleeding and/or resulting in organ failure) was included as an adverse maternal outcome (Table 1).

**Table 2:** Trends in maternal population characteristics, NSW 1999–2004

**Maternal and pregnancy characteristics**	**1999 N = 84934%**	**2004 N = 81381**	Change in rate relative to 1999%(95%CI)*
Maternal age			
<20 years	4.8	4.0	-15.5 (-19.6, -11.4)
20–34 years	78.2	76.0	-2.8 (-3.3, -2.3)
≥ 35 years	17.0	19.9	+17.0 (+14.8, +19.2)
			
Parity			
0	41.2	42.5	+ 3.3 (+ 2.2, + 4.5)
1–3	55.4	54.0	-2.5 (-3.4, -1.6)
≥ 4	3.5	3.5	-
			
Smoking	18.9	14.8	-21.9 (-23.8, -20.0)
			
Delivery hospital (level)			
Small rural	12.1	10.2	-15.6 (-18.1, -13.1)
District	29.4	25.8	-12.3 (-13.8, -10.8)
Tertiary obstetric	40.2	39.6	-1.3 (-2.5, -0.1)
Private	18.3	24.4	+33.0 (+30.8, +35.1)
			
Multiple pregnancy	1.5	1.6	-
			
Previous caesarean birth†	18.3	22.6	+23.7 (+20.9, +26.4)
			
Hypertensive disorders	10.9	8.9	-18.1 (-20.7, -15.5)
Preexisting hypertension	0.6	0.6	+12.3 (+ 8.1, +25.4)
Pregnancy Hypertension	10.3	8.2	-20.2 (-22.9, -17.5)
			
Diabetes during pregnancy	4.3	4.9	+13.9 (+ 9.2, +18.6)
Pre-existing diabetes	0.3	0.4	-
Gestational diabetes	4.0	4.6	+13.5 (+ 8.6, +18.3)
			
Antepartum haemorrhage	1.6	1.8	+11.7 (+ 4.0, +19.3)
			
Induction of labour‡	26.8	28.8	+7.5 (+ 5.7, + 9.2)
			
Mode of delivery			
Spontaneous vaginal	69.4	62.4	-10.0 (-10.7, -9.4)
Instrumental	10.9	10.3	-5.8 (-8.5, -3.1)
Caesarean section	19.7	27.3	+38.5 (+36.4, +40.5)
			
Postpartum haemorrhage	6.2	6.8	+10.7 (+ 6.9, +14.6)

* Changes with a significant trend over time χ^2 ^for trend P < 0.01 are reported.

† among who had prior births.

‡ Excludes caesarean sections prior to labour.

**Table 3:** Risk factors for adverse maternal outcome among 31,269 women with a postpartum haemorrhage

	**Maternal morbidity**			
	**Yes N = 3745%**	**No N = 27524%**	**Crude OR (95%CI)**	Adjusted OR* (95%CI)
Year (ref = 1999) †			1.03 (1.01–1.05)	1.03 (1.01–1.05)
Maternal age				
<20	4.9	4.2	1.24 (1.05–1.45)	1.25 (1.06–1.48)
20–34	73.6	78.3	1.00	1.00 (Referent)
≥ 35	21.5	17.5	1.30 (1.19–1.42)	1.16 (1.06–1.27)
Parity				
0	48.8	47.1	1.18 (1.09–1.28)	1.14 (1.04–1.25)
1	26.5	30.3	1	1.00 (Referent)
2	13.4	13.6	1.12 (1.00–1.28)	1.10 (0.98–1.23)
3	5.9	5.3	1.24 (1.06–1.45)	1.19 (1.01–1.40)
≥ 4	5.6	3.8	1.69 (1.43–1.99)	1.41 (1.19–1.68)
Previous caesarean	12.1	6.9	1.85 (1.66–2.07)	1.52 (1.33–1.73)
Multiple pregnancy	5.3	2.6	2.12 (1.80–2.49)	1.61 (1.35–1.92)
Malpresentation	5.3	3.1	1.68 (1.43–1.97)	Not retained
Smoking	17.8	15.2	1.21 (1.10–1.32)	1.19 (1.08–1.31)
Diabetes				
Pre-gestational	0.5	0.3	1.76 (1.04–2.99)	Not retained
Gestational	5.3	4.4	1.19 (1.02–1.39)	Not retained
Hypertension	16.3	11.6	1.49 (1.35–1.64)	1.30 (1.18–1.44)
Renal disease	0.4	0.1	2.92 (1.61–5.32)	2.76 (1.49–5.11)
Cardiac disease	1.7	0.3	5.10 (3.67–7.10)	4.13 (2.94–5.81)
Antepartum haemorrhage	8.3	2.5	3.54 (3.07–4.07)	2.53 (2.16–2.95)
Hospital type				
Small rural	10.8	9.1	1.22 (1.09–1.37)	1.41 (1.26–1.59)
District	27.0	26.3	1.06 (0.97–1.15)	1.18 (1.08–1.28)
Tertiary obstetric	49.5	50.9	1.00 (Referent)	1.00 (Referent)
Private	12.7	13.7	0.96 (0.86–1.07)	0.99 (1.88–1.09)
Induction of labour	33.1	30.1	1.15 (1.07–1.24)	1.19 (1.10–1.28)
Mode of delivery				
Spontaneous vaginal	54.8	70.3	1.00 (Referent)	1.00 (Referent)
Instrumental	21.1	17.2	1.59 (1.45–1.73)	1.56 (1.42–1.72)
CS prior to labour	11.4	5.7	2.60 (2.31–2.92)	1.74 (1.51–2.00)
CS during labour	12.7	6.8	2.38 (2.13–2.66)	1.99 (1.77–2.24)
Gestational age (weeks)				
20–32	3.8	1.9	2.05 (1.68–2.49)	1.39 (1.13–1.72)
33–36	6.9	4.0	1.80 (1.56–2.08)	1.27 (1.09–1.48)
37+	89.3	94.1	1.00 (Referent)	1.00 (Referent)

*Adjusted for other factors in the table as indicated; Hosmer-Lemeshow Goodness-of-Fit

P = 0.46.

† linear, for each extra year the odds ratio (OR) approximates change in rate.

CS caesarean section.

### Analyses

First we examined changes in the frequency of maternal and pregnancy characteristics from 1999 to 2004. For characteristics that changed significantly over time (χ^2 ^for linear trend P < 0.01) we calculated the absolute change in the rate in 2004 relative to 1999. We determined the rate of adverse maternal outcomes, both overall and among women with PPH, per 1000 women giving birth.

We developed two logistic regression models for adverse maternal outcomes, one among all women and one among only those with PPH. The aim of the first model was to examine whether any change in the rate of adverse outcomes over time was due solely to changes in known pregnancy and birth factors. As severe adverse maternal outcomes are rare, annual change in the rate from 1999 to 2004 was estimated from the odds ratio (OR) for 'year' derived from this first logistic regression model. The aim of the second model was to examine risk factors for adverse maternal outcome among women with PPH and whether they accounted for changes over time. Pregnancy and birth factors were included in both initial models if the Wald chi-squared test gave P < 0.1. Least significant factors were progressively eliminated from each initial model, only being retained if they had P < 0.01 or if they were confounders (change in OR of 10% or more). Crude and adjusted odds ratios (aOR) and 95 percent confidence intervals (95% CI) were calculated from the regression coefficients and their standard errors. Although the models include some factors that may lie on the causal pathway to adverse outcomes, (e.g. previous caesarean section, antepartum haemorrhage due to placenta praevia and caesarean section in the index pregnancy [26]), the causal pathways are multifactorial and the factors are also independent risk factors for adverse outcomes. As our aim was to see if changes in any of the risk factors accounted for changes in adverse outcomes over time, we chose an inclusive model. Consequently, the adjusted odds ratios for the more distal risk factors may be under-estimated.

Finally, we examined the impact of adverse maternal outcomes on maternal length of stay during the birth admission as a measure of health service impact. We determined the total number of hospital bed days for women with and without an adverse outcome and calculated the absolute and percentage change over the study period. The study was approved by the Sydney South West Area Health Service Human Research Ethics Committee and the University of Sydney Human Ethics Research Committee.

## Results

From 1999 to 2004, the number of women giving birth in NSW decreased by 4.2%, from 84,934 in to 81,381. There were also significant changes in the characteristics of women giving birth during the study period, including some which are recognised risk factors for adverse maternal outcomes (Table 2). For example there were significant increases in women aged ≥ 35 years, women having first births, and women with a prior caesarean section, and in antepartum and postpartum haemorrhage rates. Births in rural and district hospitals declined, as did the average length of the birth admission from 4.3 to 3.4 days.

Of the 500,603 women giving birth between 1999 and 2004, the MMOI identified 6242 (12.5 per 1000) as suffering severe adverse outcomes, including 22 women who died in hospital. The MMOI component diagnoses and procedures and their rates per 10,000 deliveries are reported in Table 1. Morbid procedures were more commonly reported than morbid diagnoses; 5359 (85.8%) women underwent one or more procedures, and 1255 (20.1%) women had at least one diagnosis indicating an adverse outcome. The majority (84.7%) had only a single morbid event or procedure reported, 661 (10.6%) had two events or procedures and 293 (4.7%) had three or more events or procedures.

The annual rate of adverse maternal outcomes increased from 11.5 per 1000 in 1999 to 13.8 per 1000 in 2004, an overall increase of 20.9% and a relative increase of 3.8% per annum (95%CI 2.3–5.3%). The pregnancy and birth factors in Table 2 did not account for this increase in adverse maternal outcomes (data not shown). The increase was primarily due to increases in transfusions of blood or blood products from 682 (8.0 per 1000) in 1999 to 870 (10.7 per 1000) in 2004, a relative increase of 5.1% per annum (95%CI 3.3%–6.9%).

**Figure 1:**
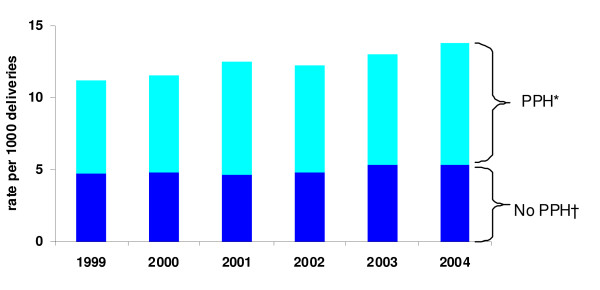
**Trend in severe adverse maternal outcomes during the birth admission, 1999–2004**. * Rate of adverse outcomes among women who had a PPH. † Rate of adverse outcomes among women who did not have a PPH.

Among the 6242 women with an adverse maternal outcome the contribution of recognised predisposing risk factors was as follows: 67% of women with an adverse outcome had an obstetric haemorrhage (60% postpartum haemorrhage and/or 13% antepartum haemorrhage during the birth admission), 18% had a hypertensive disorder, 6.1% diabetes, 3.8% cardiac disease, 0.7% renal disease and/or 1.9% sepsis. The overall increase in adverse maternal outcomes occurred almost entirely among women who had a PPH (Figure 1). Although adverse outcomes also increased among women with hypertensive diseases (from 2.0 to 3.0%), over half these women also had a PPH.

The rate of PPH increased significantly from 6.2% in 1999 to 6.8% in 2004 (Table 2). Most women with a PPH were managed without the need for a transfusion or procedure to control bleeding and hence were not considered to suffer a severe PPH. Among women with PPH the rate of adverse outcomes increased from 10.9% to 12.5%, an overall increase of 14.3% and a relative increase of 3.1% per annum (95%CI 1.2%–5.2%). For these women with PPH, the risk factors associated with a adverse maternal outcome in both univariable and multivariable analyses included: maternal age <20 or ≥ 35 years, extremes of parity, previous caesarean section, multiple pregnancy, smoking during pregnancy, maternal medical conditions, antepartum haemorrhage and induction of labour and birth in a small rural hospital (Table 3). Factors that were not predictive of adverse outcome among women with a PPH and were excluded from the multivariable analysis included gestation at first antenatal visit, augmentation of labour, perineal tears, episiotomy and regional analgesia.

Overall, the decline in the number of births and in the duration of hospital admissions resulted in a net decline of almost 85,000 annual maternal hospital days for births from 1999 to 2004 (Table 4). Among women with a severe adverse outcome together with a PPH there was a 7% decrease in hospital days over the study period, whereas women with a PPH but no severe adverse outcome occupied 21% fewer hospital days in 2004 than in 1999. The relative decline in length of stay for women without adverse outcomes was similar irrespective of whether they had a PPH (Table 4).

**Table 4:** Decline in duration of the birth admission in NSW from 1999 to 2004, by occurrence of adverse maternal outcome and postpartum haemorrhage (PPH)

	**Total length of stay (days)**		
	**1999 (84,934 women)**	**2004 (81,381 women)**	**Decline in length of stay 1999–2004 No of days (% change)**
All women	364,820	280,238	-84,582 (-23.2%)
No severe morbidity	357,745	274,039	-83,706 (-23.4%)
PPH, no severe morbidity	21,220	16,812	-4,408 (-20.8%)
Any severe morbidity	7,075	6,199	-876 (-12.4%)
PPH with severe morbidity	3,782	3,508	-274 (-7.2%)

## Discussion

Our results indicate that adverse maternal outcomes associated with childbirth are increasing in an Australian population and that the increase was almost entirely among women who experienced a PPH. Significant changes in the characteristics of women giving birth, and in obstetric practice, did not explain the increase in severe adverse maternal outcomes.

Internationally, studies of maternal morbidity during the birth admission have reported incidence rates ranging from 3.8 to 430 per 1000 deliveries [4,7,8,12,16,27]. Direct comparison is hampered by different definitions, methods of case finding and selection of study populations. The study with a very high morbidity rate (430/1000) was one where anything other than a normal delivery was considered a maternal morbidity including pre-existing medical conditions and caesarean delivery [27]. Our intention was to capture, at a whole population level, women who suffered potentially preventable adverse outcomes, including 'near miss' events, as a measure of the quality of care. We used an outcome indicator that would occur with sufficient frequency to avoid random fluctuations and be detectable in low-volume hospitals [11]. Like others we have chosen to measure adverse outcomes and not the factors that predispose to them [4,8,12,16]. For example, although severe preeclampsia may result in significant adverse outcomes, it can also be well managed without adverse maternal outcome.

The increase in maternal morbidity in this study was attributable to the 32% increase in blood transfusions. This is consistent with the findings of US and Scottish studies examining trends in severe maternal morbidity [4,12]. Callaghan and colleagues found that maternal morbidity in the US increased by 31% from 1991 to 2003, attributable to an increase in blood transfusions [4]. In Scotland, severe maternal morbidity increased by 17% from 2003 to 2005, and the increase was almost entirely accounted for by an increase in major obstetric haemorrhage [12]. In contrast, a Canadian study found a stable rate of maternal morbidity between 1991 and 2000 which occurred in the context of comparatively low and stable rates of postpartum haemorrhage with transfusion [8,14].

Callaghan et al considered the possibility that increasing transfusion rates may reflect a more permissive attitude towards blood transfusions among obstetricians or differences in medical record coding practice [4]. Rates of transfusion among women with a PPH increased 5-fold in NSW during the 1990s and a more permissive attitude was considered a possible explanation for the increase [13,28]. However, the publication of [Australian] Clinical Practice Guideline on the Use of Blood Components in 2001 and initiatives to improve the appropriateness of red cell transfusions has anecdotally resulted in a less permissive attitude towards blood transfusion [29]. A limitation of hospital discharge data is that the number of units transfused is not available and so trends cannot be explored. We consider changes in reporting to be an unlikely explanation for the increase in transfusions in Australia. Validation studies have found transfusion reporting is accurate, has ascertainment around 85% and has not changed over time [11,30]. Similarly, PPH reporting has not changed over time, with ascertainment around 74% overall and 93% for PPH requiring transfusion [21,31]. However, it has also been suggested that there is systematic under-reporting of haemorrhage following caesarean section.

If clinicians have been more reluctant to use blood transfusions, our results suggest that not only is the PPH rate increasing but so is the severity of the haemorrhage. Risk factors for PPH have been thoroughly documented and two studies have investigated the increasing rates [14,28]. Both studies found that the increase in PPH rates is not explained by changes in known risk factors including increasing maternal age, multiple pregnancies, caesarean sections, placenta praevia, induction and augmentation of labour, prolonged second stage of labour and fetal size [14,28]. These population-based studies lacked information on some obstetric practices, such as management of the third stage of labour and monitoring in the early postpartum period, and inadequacy of these practices may be an explanation for the increase in PPH.

Active management of the third stage of labour is effective in reducing PPH and the burden of disease associated with haemorrhage [32]. The International Confederation of Midwives and the International Federation of Gynecologists and Obstetricians (ICM/FIGO) recommend active management of the third stage of labor for all women [33]. However, adherence with active third-stage management recommendations is poorly reported and/or suboptimal in Australia, and significant variations in policies and practice have been reported elsewhere [33-35]. Suboptimal adherence with active management guidelines could explain rising PPH rates.

We identified a number of risk factors for severe adverse maternal outcomes among women who suffer a PPH, although the effect of the more distal risk factors, such as prior caesarean section, may be under-estimated (see methods). Factors that commonly occur in the population and have a moderate risk (eg induction of labour and operative deliveries) will make a greater population contribution to adverse outcomes than rare exposures with markedly elevated risks (eg renal and cardiac disease). The non-specific nature of the risk factors for adverse maternal outcomes among women with a PPH reinforces the proposition that management and monitoring protocols for the early identification and prevention of PPH should focus on all women not just those considered to be at risk [36].

The risk factor for PPH-associated adverse outcomes that is most amenable to intervention is place of birth. After adjusting for casemix, women with a PPH at a small rural hospital were over 40% more likely to suffer an adverse maternal outcome than women delivering at a tertiary obstetric hospital. Maternity services in rural areas need to be resourced with adequate staff, skills and facilities to manage PPH. A survey of the uptake of a PPH prevention and management policy in NSW reported inadequate postpartum monitoring (blood loss, fundal tone, pulse, blood pressure) of women, especially in small rural hospitals, and that staff shortages were a barrier to implementing the policy[37] ICM/FIGO recommends careful observation and monitoring every 15 minutes during the first 2 hours following delivery, with palpation of the uterus (and massage if necessary) [36]. In view of the potential hazard of bleeding due to uterine atony, genital tract trauma and retained products of conception, there is an urgent need to standardise, implement and resource a policy of careful observation and monitoring in the 2-hour period following delivery.

Whilst all women should be regarded as being at risk of haemorrhage, our findings suggest that some women can be identified antenatally (eg those with prior caesarean section, multiple pregnancy, renal or cardiac disease) as having a substantially increased relative risk of an adverse outcome if a PPH occurs, and these risk factors merit consideration of a higher level of obstetric care for delivery and increased vigilance postpartum.

The short and long term consequences of adverse maternal outcomes can be profound including surgery, emergency care, infertility, psychological effects, disability and even death. Although the number of days in hospital represent the 'tip of the iceberg' for costs to women and the health system, they provide a snapshot of the impact of adverse maternal outcomes on the health system. Overall the number of days spent in hospital at the time of delivery declined by 23%, although the number of women giving birth decreased by only 4.2%. For women with a severe adverse outcome, however, the decline in bed-days was much lower (12% overall and 7% among those with PPH) indicating that the impact of adverse maternal outcomes are relatively intractable and that costs could be better reduced by prevention than improved management after the event.

The major strength of this population-based cohort study is the use of outcome and exposure measures that are accurately and reliably reported in population health data. The development and validation of the MMOI has been described in detail elsewhere [11]. Briefly, the study had three phases: first conditions and procedures that could comprise a severe maternal morbidity indicator were catalogued by reviewing the literature and consulting with clinicians; second, was a validation study of the initial indicator by review of the medical records; and finally adverse outcomes, as determined from the medical record review, was used to refine the initial indicator to give an MMOI that identified severe adverse maternal outcomes from the population health data with a high positive predictive value (95%). Although the number of available diagnosis or procedure fields in each medical record increased over the study period, the MMOI was applied to the same number of fields each year to ensure that any increase in adverse maternal outcome was not attributable to greater ascertainment. Furthermore, the availability of linked birth and hospital data obviates the need for complex algorithms to identify birth admissions in hospital discharge data [38].

As this study is limited to the birth admission, and does not include postpartum admissions, it could result in under-ascertainment of adverse maternal outcomes. Because the timing of events during the hospital admission can generally not be obtained from hospital discharge data, causal pathways to adverse maternal outcome may also be uncertain. However, haemorrhage is consistently reported as the largest and most important cause of maternal morbidity [4,10-12].

Caution is needed in interpreting the incidence of the individual components of the MMOI. A significant advantage of a composite outcome indicator is that it helps to overcome the recognised under-ascertainment of individual diagnoses and procedures in routinely collected data [11]. For example, we have found under-reporting of caesarean hysterectomy in hospital data, possibly because the procedure is rarely a planned procedure [11]. However, women requiring a hysterectomy to control bleeding usually receive a blood transfusion and/or other procedures to control bleeding [11,39]. These procedures are well ascertained and as long as one is recorded, these women will be indentified by the MMOI.

## Conclusion

In summary, we found that 1 in 80 women giving birth in Australia suffered a severe adverse outcome during childbirth and this rate rose to 1 in 8 women who had a PPH. Reducing or stabilising the increasing PPH rates would halt the increase in maternal morbidity. Ensuring that all women who give birth have access to active management of the third stage of labour and careful observation in the first 2 hours after delivery may reduce the PPH rate and the potential for severe morbidity and death.

## Competing interests

The authors declare that they have no competing interests.

## Authors' contributions

CLR conceived the study, and participated in its design and analysis, and drafted the manuscript. JBF, CSA and JCB participated in the study design, data preparation and analysis, and helped to draft the manuscript. JMS provided statistical expertise and critical review of the manuscript. JMM provided clinical expertise, participated in the study design and coordination of the study. All authors read and approved the final manuscript.

## Pre-publication history

The pre-publication history for this paper can be accessed here:


